# Determining Complex Structures using Docking Method with Single Particle Scattering Data

**DOI:** 10.3389/fmolb.2017.00023

**Published:** 2017-04-25

**Authors:** Hongxiao Wang, Haiguang Liu

**Affiliations:** Complex Systems Division, Beijing Computational Science Research CenterBeijing, China

**Keywords:** hybrid method, single particle scattering, x-ray free electron laser, docking, molecular complex

## Abstract

Protein complexes are critical for many molecular functions. Due to intrinsic flexibility and dynamics of complexes, their structures are more difficult to determine using conventional experimental methods, in contrast to individual subunits. One of the major challenges is the crystallization of protein complexes. Using X-ray free electron lasers (XFELs), it is possible to collect scattering signals from non-crystalline protein complexes, but data interpretation is more difficult because of unknown orientations. Here, we propose a hybrid approach to determine protein complex structures by combining XFEL single particle scattering data with computational docking methods. Using simulations data, we demonstrate that a small set of single particle scattering data collected at random orientations can be used to distinguish the native complex structure from the decoys generated using docking algorithms. The results also indicate that a small set of single particle scattering data is superior to spherically averaged intensity profile in distinguishing complex structures. Given the fact that XFEL experimental data are difficult to acquire and at low abundance, this hybrid approach should find wide applications in data interpretations.

## Introduction

In crowded cellular environment, protein molecules often form complexes to fulfill their functions. Thus, the study of protein complex structures and dynamics is critical for the understanding of molecular mechanism (Eisenberg et al., 2000; Bader et al., 2003; Krissinel and Henrick, 2007). Because protein complexes are mostly stabilized by non-covalent interactions, their stability is under strong influence of solvent conditions, making it difficult to form molecular crystals that can yield strong diffraction signals. The nuclear magnetic resonance (NMR) spectroscopy has been widely applied to structure determination of relatively small molecular systems, but the degeneracy of NMR signals in large protein complexes challenges the model reconstructions (Bax and Grzesiek, 1993; Mainz et al., 2013; Göbl et al., 2014; Shen and Bax, 2015). Other experimental approaches that do not require crystallization include small angle X-ray scattering (SAXS) methods that obtain rotational averaged scattering intensity profile, from which structural information can be extracted to build low resolution 3D models (Konarev et al., 2006; Liu et al., 2012). Biochemistry techniques, such as cross-linking, mutagenesis, or single molecule fluorescence experiments can reveal critical interacting regions at complex interfaces, for example. The SAXS and biochemistry assay data bear a common problem: the information deficiency, compared to X-ray crystallography or NMR, does not allow a high resolution 3D structure determination. The data interpretation therefore heavily depends on computational modeling.

Recent advances in single particle imaging (SPI) methods using cryogenic electron microscopy (cryo-EM) or the emerging X-ray Free Electron Laser (XFEL) provide a new opportunity to study the molecular complex structure and dynamics (Emma et al., 2010; Chapman et al., 2011; Seibert et al., 2011; Cheng, 2015; Cheng et al., 2015; Schlichting, 2015). The cryo-EM single particle imaging technology has achieved significant breakthroughs, mostly thanks to the development of direct electron detecting device, model reconstruction algorithms, and sample handling, and automated data collection (Scheres, 2012; Cheng, 2015; Cheng et al., 2015). The resolution of 3D reconstruction models from cryo-EM data has been reported to atomic resolution, and the molecular size can be smaller than 100 kDa (Merk et al., 2016). The XFELs with their unprecedented peak brilliance realized a new experimental mode, “diffract before damage,” to overcome the X-ray dosage limitations, making it possible to collect high resolution X-ray diffraction signals from non-crystal single molecule samples in principle (Neutze et al., 2000; Bogan et al., 2008; Seibert et al., 2011; Munke et al., 2016). Since the commissioning of the world's first hard XFEL facility, the Linac Coherent Light Source (LCLS), collective efforts have been made to push forward the application of XFEL in structure determination using single particle diffraction approach, and progress has been achieved toward high resolution structure determinations (Aquila et al., 2015; Munke et al., 2016). Nevertheless, both cryo-EM single particle imaging and XFEL single particle diffraction require tremendous amount of data measured at orientations that span SO(3) rotation space to assemble into a finely sampled 3D diffraction volume, from which 3D structures can be reconstructed. It is still a limiting step to obtain such experimental datasets, especially for XFEL single particle diffraction cases (Aquila et al., 2015). Experimental challenges include sample purification, injection, and alignment to the X-ray incidence beam etc., making the data collection very tedious and inefficient. Because of the low hit-rate (the chance for XFEL pulses hitting on individual clean sample particle) and the limited XFEL resources all over the world (only LCLS in SLAC national laboratory and the SACLA in RIKEN SPring-8 center are currently commissioned), collecting a full dataset which may include millions of single particle scattering patterns is still beyond present reach as routine experiments. Therefore, the data analysis methods in cryo-EM single particle imaging is not yet practical for XFEL single particle scattering data interpretation. The computational challenges for XFEL data analysis are summarized in a recent review (Liu and Spence, 2016).

Computational docking methods have been developed for protein complex structure prediction based on the structures of protein subunits. The Critical Assessment of PRedicted Interactions (CAPRI) contests have been organized and progress has been reported in the proceedings published after each evaluation (Janin, 2005; Lensink et al., 2016). One of the major challenges in protein complex structure prediction is to design reliable scoring functions for model quality assessment. The scoring functions for docking usually incorporate the following terms to rank the predicted models: the shape complementary between protein subunits, electrostatic interactions, solvation energy, and statistical potential energy derived from protein structure databases. Although encourage progress is obtained, a satisfactory scoring function is still needed (Gray et al., 2003; Vreven et al., 2015). The aforementioned XFEL single particle scattering data can be valuable in improving the ranking of protein complex structures generated using docking method, even for the cases that the dataset is not sufficient for high resolution structure determination. As a matter of fact, similar ideas have been implemented for SAXS data, which can be incorporated in model evaluation (Mattinen et al., 2002; Zheng and Doniach, 2002; Förster et al., 2008; Schneidman-Duhovny et al., 2012; Schindler et al., 2016). In this work, we extend this approach to XFEL single particle scattering data, inspired by the application of XFEL data in modeling of protein conformation changes (Tokuhisa et al., 2016). Using Zdock program(Chen et al., 2003), structure decoy sets are generated for several selected protein complexes, and the power of ranking using the original Zdock score, the SAXS score, and the single particle scattering score is studied. The simulation results suggest that the XFEL single particle data has the most information that best distinguish the correct models from the rest in the decoy sets. The problems in experimental data based model selection and the challenges in scoring function calculation are discussed.

## Methods

### Single particle scattering pattern simulations

The scattering pattern simulation for a given protein structure is a forward problem, which is straightforward by using the Fourier transform of the electron density represented with atomic positions. In this work, the structural form factors ***F***(***q***) is calculated using the direct summation of scattered wavefunctions, i.e.,

(1) $$F(q)\;=\;\sum_{j}f_{j}(q)e^{iq\;\cdot \;r_{j}}$$

where the ***q*** is a vector in Fourier space, corresponding to the momentum transfer of the X-rays, defined as ***q*** = **2π**(***K***_**0**_ – ***K***_**i**_), ***K***_**i**_ and ***K***_**0**_ are the incidence and scattered wave vectors. *f*_*i*_(*q*) and ***r***_*j*_ are the form factor and position of atom *j*. The atomic form factor depends on the magnitude of momentum transfer q = |q|; the values can be looked up in the International Table for Crystallography. For a forward scattering experiment, the momentum transfer q can be calculated as

(2) $$q\;=\;\frac{4\;\pi \sin\theta }{\lambda }$$

and **2θ** is the scattering angle that can be calculated based on the distance between sample and detector and the pixel location information, λ is the wavelength of X-rays. Based on the construction of Ewald sphere, for a given model at any specified orientation, the structure form factor ***F***(***q***) at momentum transfer ***q*** that is mapped to the pixel position on 2D detector can be calculated using Equation (1). Then the squared modulus of the structure factors is taken for scattering intensity, i.e., *I***(q)** = ||*F***(q)**||^2^. For experimental data, Poisson noise was added to simulate the statistics error occurred during photon detection. On top of this, background noise was simulated by adding random photons following a Gaussian distribution at desired noise levels.

The key parameters for the pattern simulations can be found in Table S1 in the Supplementary Material. Experimental scattering intensity is proportional to the incidence beam intensity ***I***_**0**_, which can be used to scale the intensity values recorded with detector. Therefore, ***I***_**0**_ in this study has an immediate impact to the resolutions of scattering signals. In the simulations presented here, the incidence beam intensity was not explicitly considered. Instead, ***I***_**0**_ was used as a scaling factor to set the highest measurable resolutions. In the simulations presented in this paper, we set the highest measurable resolution shell to be 4Å, where the average number of photons recorded at each pixel in this resolution is 1. This requires the photon flux is 1–2 order of magnitudes higher than the current XFELs, such as the LCLS, whose photon flux is about 10^12^ photons/pulse/μm^2^.

The patterns for the native structures of the complexes are first simulated at random orientations in SO(3) rotation space (or a subspace) as the “experimental data”; then the patterns for the predicted models are generated with two orientation sampling approaches: (1) using the *same* orientations as the “experimental data” to study the ranking power of the scoring functions under ideal situations; and (2) using orientations specified by Euler angles spanning SO(3) rotation space. In the latter case, the orientations will be determined by computing the cross-correlation between “experimental patterns” and “model patterns,” therefore the discretizing step size is important for finding the correctly matched orientations. All patterns are simulated to 4 Å resolution.

### Protein complex generation using Z-dock program

The protein complex structures were generated using the Z-dock program developed by Weng's group in University of Massachusetts. Using Z-dock program, protein complexes were generated and 1,000 structures with high Z-dock scores were saved for single particle scattering pattern simulations. The root-mean-square-deviation (RMSD) values of these predicted models compared to the native (correct) complex structure are also recorded.

### Scoring function based on X-ray scattering data

The scoring function for Z-dock program is based on molecular shape complementary, electrostatic interaction, and solvation energy etc. Higher scores indicate better chance to be the correct model. With simulated X-ray scattering data, the chi-score is used to measure the difference between datasets to reflect the structural differences. For single particle scattering data composed of *N* scattering patterns, having intensity values in *M* pixels, the SPI chi-score is defined as:

(3) $$\chi_{\text{spi}}^{2}\;=\;\frac{1}{N}\sum_{n\;=\;1}^{N}\frac{1}{M}{\sum_{m\;=\;1}^{M}\left(\frac{I_{model}^{\left(n,m\right)}-I_{data}^{\left(n,m\right)}}{\sigma_{data}^{\left(n,m\right)}}\right)}^{2}$$

where *I*^(*n, m*)^ is the intensity value in *n-th* pattern at pixel position *m*, and σ^(*n, m*)^ is the associated standard deviation in the simulation data, σ^(*n, m*)^ = (*I*^(*n, m*)^)^1/2^according to the Poisson noise distribution. The subscripts, *model* and *data*, refer to the values corresponding to the structures generated by Z-dock, and the values corresponding to the correct model (*data* means the simulated experimental data; while *model* means the theoretical value calculated from the predicted models). Note that the *n-th* model pattern must be in the same orientation as the *n-th* “experimental” pattern for Equation (3) to be valid. In reality, orientation is unknown during the chi-score calculation for real experimental data. Therefore, orientation matching must be carried out by minimizing the chi-score for each experimental pattern with respect to all possible orientations of the model. The Equation (3) becomes:

(4) $$\chi_{\text{spi}}^{2}\;=\;\frac{1}{N}\sum_{n\;=\;1}^{N}\underset{n^{\prime }}{\min}\left(\frac{1}{M}\sum_{m\;=\;1}^{M}{\left(\frac{I_{model}^{\left(n^{\prime },m\right)}-I_{data}^{\left(n,m\right)}}{\sigma_{data}^{\left(n,m\right)}}\right)}^{2}\right)$$

where {*n*′} is the set of patterns computed for any predicted model. For finer sampled orientation space using discretized euler angles, the number of model patterns grows rapidly, so the pair-wise orientation matching is very time consuming, and we offer a possible remedy in the following sub-section.

Instead of comparing single particle patterns at matched orientations, the SAXS profiles can be obtained from experiments, or from the virtual “SAXS” pattern by summing the single particle patterns. Specifically, SAXS profile is obtained by aggregating the single particle scattering data, then averaging over the angular direction, i.e.,

(5) $$\begin{array}{l} I_{\text{SAXS}}(q)\;=\frac{1}{N}\sum_{n\;=\;1}^{N}\frac{\int_{\phi \;=\;0}^{2\pi }I^{n}(q,\phi )d\phi }{\int_{\phi \;=\;0}^{2\pi }d\phi }\\ \;=\frac{1}{2\pi N}\sum_{n\;=\;1}^{N}\int_{\phi \;=\;0}^{2\pi }I^{n}(q,\phi )d\phi \end{array}$$

*I*^*n*^(*q*,ϕ) is the intensity value at polar coordinate (*q*,ϕ) specified by the radial component *q* and the azimuth angle ϕ for the *n-th* pattern. The chi-score can be calculated as:

(6) $$\chi_{\text{SAXS}}^{2}\;=\;\frac{1}{K}\sum_{k\;=\;1}^{K}{\left(\frac{I_{\text{SAXS},\text{ model}}(q_{k})-I_{\text{SAXS},\text{ data}}(q_{k})}{\sigma_{\text{SAXS},\text{ data}}(q_{k})}\right)}^{2}$$

### Orientation matching

In order to find the orientation that best matches each “experimental” pattern, it is necessary to generate an orientation grid that spans SO(3) rotation space by discretizing three Euler angles. The step size for discretization is critical to the accuracy of orientation match. The step size can be estimated by matching the highest resolutions of 2D scattering patterns.

In order to find the best matched orientations, theoretical patterns must be simulated for all discretized orientations (after removing symmetric redundancies if there are any). Then each “experimental” pattern must be compared to all theoretical patterns for the theoretical model. The best matched pattern is identified by finding the lowest chi-scores compared to each experimental pattern. It is very computational expensive to evaluate chi-scores for all “experiment-model” pattern pairs at pixel levels. For example, if each rotational Euler angle is discretized to *n* values, to find orientations of *m* experimental patterns, there will be *n*^*3*^*m* evaluations of 2D matrix comparison. This computational challenging problem can be sorted out in several approaches, and here we offer two solutions.

First, for the simulation case, as a proof-of-principle, we artificially confine our rotational degree of freedom within a subspace of SO(3) defined by the Euler angles (−22.5° ≤ α,β,γ ≤ 22.5°). This does not solve the problem in actual applications to experimental data, which are certainly not confined to this subspace, yet this operation allows quick assessment of the effects of grid size.

The second solution is to reduce the “experimental” pattern to its angular auto-correlation, which does not depend on the in-plane rotation angle (Kam, 1977; Liu et al., 2013; Huang and Liu, 2016). The angular auto-correlation function (AC) is defined as:

(7) $$AC(q,\Delta \phi )\;=\;\int_{0}^{2\pi }I(q,\phi )I(q,\phi +\Delta \phi )d\phi$$

where *I(q*,ϕ*)* is the intensity at pixel specified using polar coordinate (*q*,ϕ). This requires a pre-processing of the “experimental” patterns and the theoretical patterns computed from predicted models. The AC transformation removes the in-plane rotation dependence of the scattering pattern, making the AC function depend on two Euler angles that specify a direction perpendicular to the scattering pattern. Then the AC functions are used for pairwise comparison for scoring (i.e., chi-scores of AC functions are calculated), rather than comparing each scattering pattern with every reference pattern. It can be shown that the extra overhead calculation has benefit in reducing the computational complexity from O(n^3^M) to O(n^2^M), where *n* is the number of grids for each Euler angle, and *M* is the number of experimental patterns. The computational complexity for overhead computing of AC function is O((n^3^+M)^*^k), where n^3^, M are the numbers of theoretical patterns and “experimental” patterns respectively, k is the number of discretization of in-plane rotation angle. The advantage is obvious if M>>k.

## Results

In this section, using simulation data with the docking decoys, we will answer four questions: (1) how many single particle scattering patterns are needed for the scoring function to converge; (2) how do the scoring functions compare to each other in terms of ranking the predicted models; (3) how does the orientation mismatching affect accuracy of the scoring functions; (4) how to speed up the orientation matching by using reduced representations of scattering patterns.

**Figure 1:**
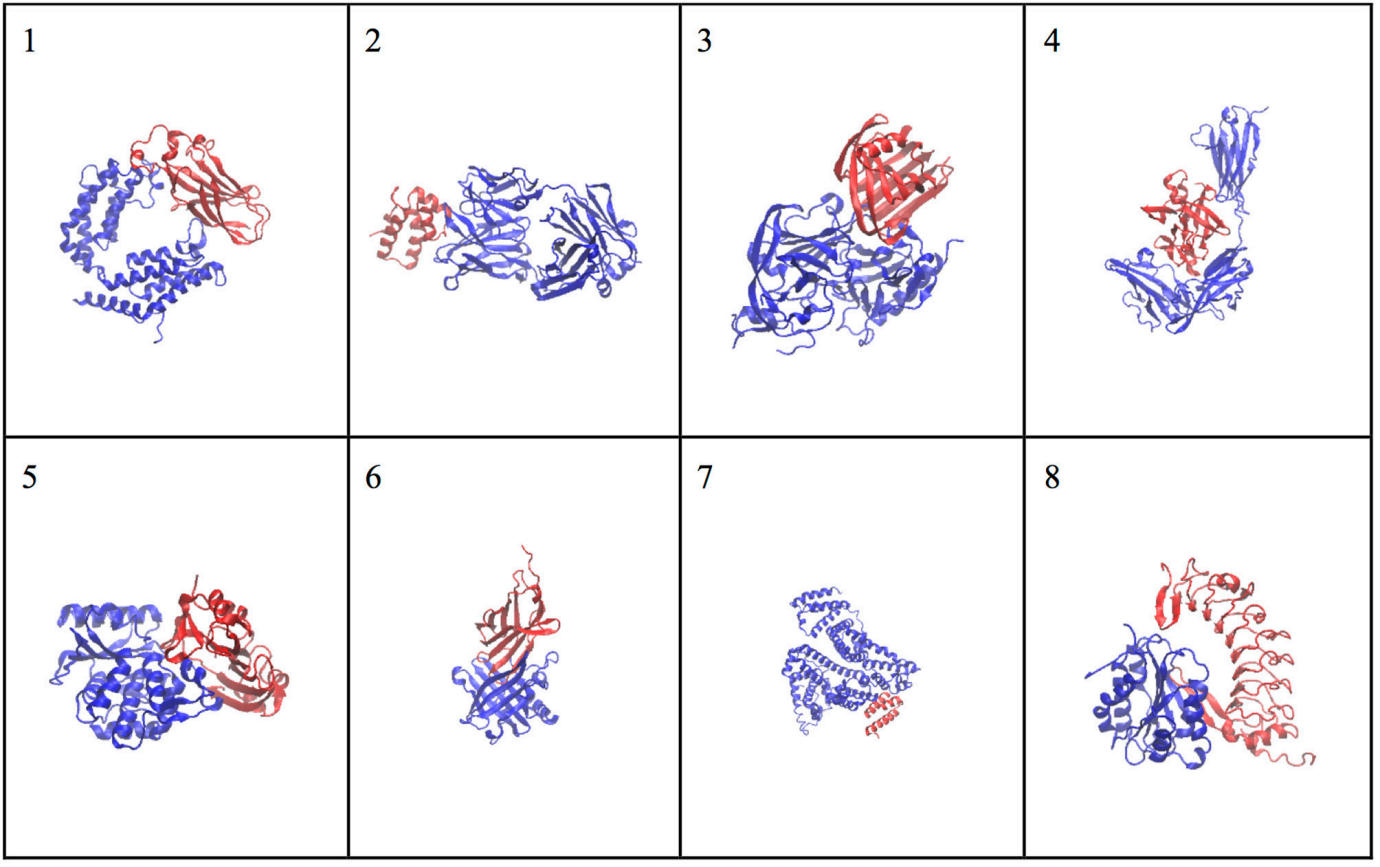
**The native structures for the molecular complexes used in this study**. Two subunits are colored in blue and red in each complex, where the blue subunit is fixed and the red subunit moves around the blue subunit to generate complex structures.

The molecular complex systems are selected from Benchmark 5.0 on Z-dock server (Vreven et al., 2015). The models are depictured in Figure 1 and major features are summarized in Table 1. The native structures are available at http://liulab.csrc.ac.cn/download/zdock/.

**Table 1:** **The characteristics of the molecular complexes**.

**ID**	**Complex PDB code**	**Subunit 1 (S1)**	**Subunit2 (S2)**	**No. atom of S1**	**No. atom of S2**	**No. Residue of S1**	**No. Residue of S2**	**Difficulty in Zdock**	**No. atom of complex**	**No. Residue of complex**
1	**3AAD_A:D**	1EQF_A	1TEY_A	2,164	1,231	243	144	Difficult	3,395	387
2	**2B42_B:A**	2DCY_A	1T6E_X	2,604	1,443	341	171	Easy	4,047	512
3	**1E6J_HL:P**	1E6O_HL	1A43_	3,275	577	397	69	Easy	3,852	466
4	**1IRA_Y:X**	1G0Y_R	1ILR_1	2,499	1,139	294	138	Difficult	3,638	432
5	**1JTG_B:A**	3GMU_B	1ZG4_A	2,021	1,234	242	155	Easy	3,255	397
6	**3BX7_A:C**	3BX8_A	3OSK_A	1,389	897	163	111	Middle	2,286	274
7	**2VDB_A:B**	3CX9_A	2J5Y_A	4,345	436	528	52	Easy	4,781	580
8	**1M10_A:B**	1AUQ_	1M0Z_B	1,601	2,087	184	254	Middle	3,688	438

*The complex structures are shown in Figure 1, labeled with the complex ID*.

### The convergence of scoring function

Both the SPI-score and SAXS-score (Equations 3, 6) need a good number of patterns to reach convergence. The first task is to determine the lower limit of this number using simulation data. Experimentally, the SAXS profile can be obtained without too much technical challenge, and even high throughput data collection is possible for standard SAXS experiments. We focus on the convergence of SPI-score in this section, because high quality single particle scattering patterns are still very difficult to obtain, even at X-ray free electron laser facilities. This is also one of the major motivations of this work, through which we hope to demonstrate that the hybrid approach for data analysis can improve the performance of both computational modeling and the XFEL data interpretation using a small set of data.

**Figure 2:**
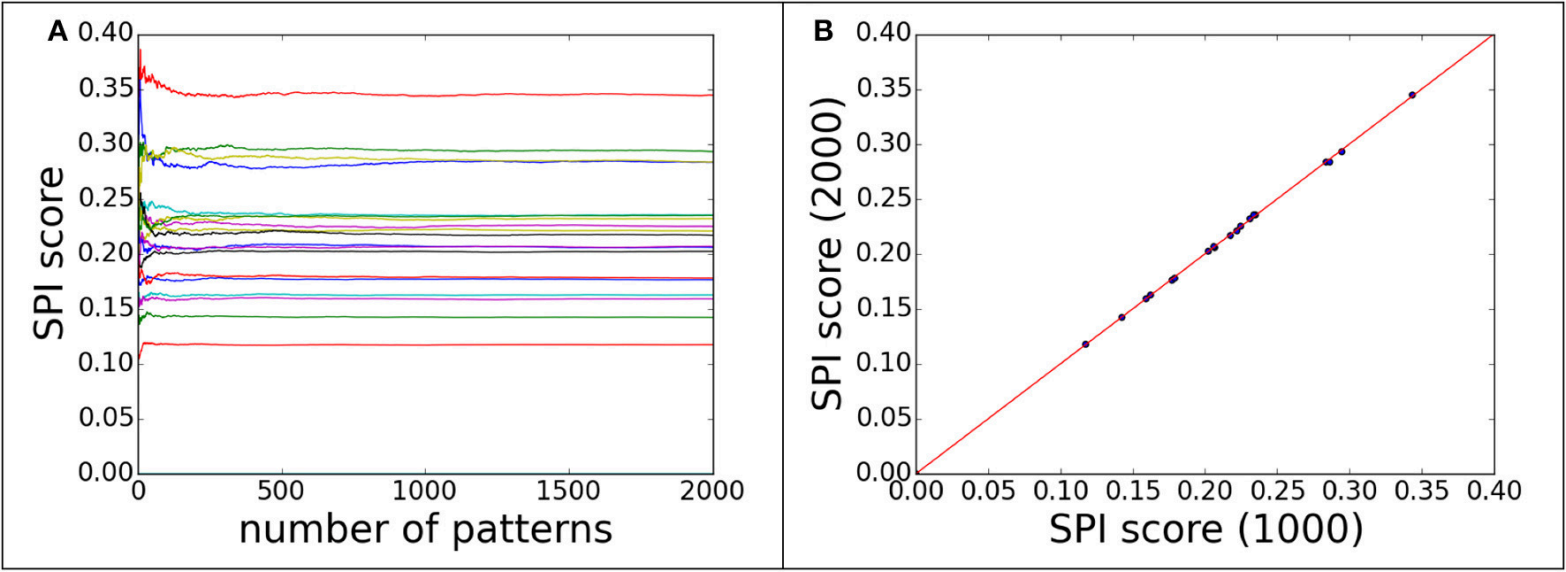
**The convergence of SPI-score for patterns with correct orientations**. 60 decoys from complex#1 are used to demonstrate the convergence progress of SPI-score. **(A)** the SPI-score is plotted as a function of pattern quantity, each line represent the SPI-score of one predicted decoy model by comparing model patterns to “experimental” data. **(B)** The comparison of SPI-scores computed using 1,000 or 2,000 scattering patterns, whose orientations are random.

Regarding the convergence question, the SPI-score was computed with different numbers of single particle scattering patterns. The convergence can be monitored by plotting SPI-score as a function of pattern numbers. The purpose of the convergence test is to ensure that the scores are consistent and independent of number of measurement. Figure 2A shows the convergence of scoring function for 60 decoy structures of complex#1 (3AAD). Here, the goal is to find the minimum number of patterns required to yield a reliable scoring function. To rule out other factors, the orientation for each pattern was taken as known information, i.e., the exactly matched orientation was used for comparison. The actual cases where orientation assignment is required are considered in the following sections. As shown in Figure 2A, the SPI-scores have large fluctuations when the number of patterns is small, then converges quickly when the number approaches 1,000. Similar trends were observed for other complexes, and for this reason we use 1,000 scattering patterns in the SPI-score calculations through the study. It is worthwhile to note that the minimum number of scattering patterns required for a converged SPI-score varies for each system, depending on complex size, binding mode, and complex structure. The number 1,000 is a compromised choice between accuracy and speed. The SPI-scores for different predicted models are well separated when the SPI-score reach convergence, indicating that the converged SPI-scores can be used to assess the quality of the molecular complexes. In Figure 2B, for each decoy model, we compared the SPI-scores with 1,000 patterns and those with 2,000 patterns, the two sets of scores are perfectly lined up around y = x. Therefore, simulation results indicate that the convergence can be reached when number of patterns is above 1,000. In other words, the minimum number of patterns required to reliably scoring the predicted models is 1,000, which is feasible with current instruments at XFEL facilities.

### The comparison of three scoring functions

**Figure 3:**
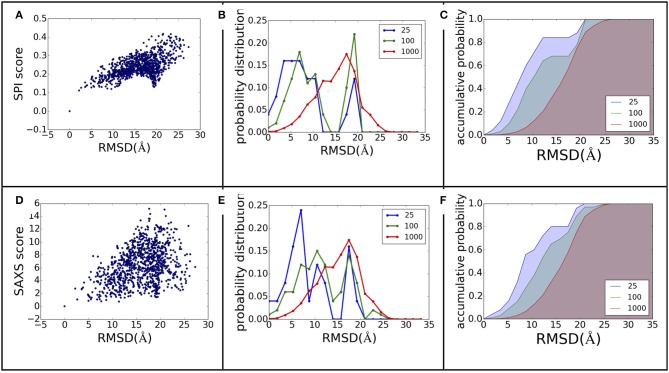
**The ranking power comparison between SPI-score and SAXS-score. (A,D)** the scatter plot of scores as a function of RMSD. **(B,E)** the probability distribution function of RMSD for the selected models. The three curves correspond to the distribution function of top 25, top 100, and all models. **(C,F)** The accumulative probability functions corresponding to the three distributions in **(B,E)**. The green and blue shaded area indicates the gain of ranking power by selecting subsets of models.

The power of ranking for each scoring function can be evaluated by studying the correlation between the scores and model differences. The RMSD is one of the most commonly used measurements for model comparison. In Figure 3, the ranking power for SPI-score and SAXS-score are summarized for the complex#1, which belongs to “difficult” docking case. As shown in Figure 3, the scattering plots clearly show that both SPI-scores and SAXS-scores are positively correlated with the RMSD values in general. For the case of complex#1, the correlation coefficients between SPI-score and RMSD is 0.59, and the correlation coefficient between SAXS-score and RMSD is smaller, giving a value of 0.36. To better quantify the ranking power of the scoring functions, a probability distribution function of RMSD, *P*^(RMSD, *n*)^, was computed for top *n* selected models. Specifically, the probability for a model differing from the native structure by a particular RMSD value was calculated for *n* models with lowest scores. The probability distribution functions are plotted in (Figures 3B,E), where the *P*^(RMSD, *n*)^ for *n* = 25, 100, 1,000 (all) are calculated and compared. Based on the probability distribution and the correlation coefficients between the scoring function and the RMSD, it is clear that both SPI-scores and SAXS-scores are capable of selecting models that have lower RMSD values with respect to the native structure, while the SPI-scores have stronger selecting power. The probability of selecting models with lower RMSD values is increased after model ranking using either SPI-score or SAXS-score. This increasing trend is more pronounced for the ranking using SPI-scores. The probability function is converted to accumulative probability function by integration, as shown in (Figures 3C,F). On the other side, the scoring function from the Z-dock can select a few best matched models from predicted models, the overall ranking power is not as good as the SPI or SAXS scoring functions (data not shown). This makes the z-dock scoring function vulnerable to insufficient model generation. The SPI-score is a more powerful function not only because it can be used to select the lowest RMSD models, but also because the model ranking is consistent with structure differences.

The same analyses were carried out for eight complexes, as described in Figure 1 and Table 1 (for the other seven complexes, see Supplementary Material). To quantify the ranking power, we define a new parameter, the area under the accumulative probability curve (AUC, area under curve), similar to the measure of classification power. For each accumulative probability distribution curve, the area is calculated by integration. The x-axis, the range of RMSD, can be normalized to the fraction of the largest RMSD value in the decoy sets. Therefore, the AUC has a largest possible area of 1.0, as an extreme case when all models are ranked in the same order as the RMSD with respect to the native structure. Under this definition, larger AUC values correspond to more powerful ranking method. We calculated AUC at three levels of selection (top 25, top 100, and all models) for each method (SPI-scoring, SAXS-scoring, and Z-dock scoring), same as the demonstration example in Figure 3. In Table 2, the AUC statistics are summarized, suggesting that SPI-score has better performance in terms of ranking power, compared to SAXS-score. There is one exception in the case of complex#2, where the ranking power of SAXS-score is slightly better than that of the SPI-score.

**Table 2:** **The performance of scoring functions**.

**Complex ID**	**Z-dock**			**SAXS**			**SPI**		
	**Top 25**	**Top 100**	**All**	**Top 25**	**Top 100**	**All**	**Top 25**	**Top 100**	**All**
1	0.53	0.55	0.55	0.71	0.64	0.54	**0.74**	0.65	0.54
2	0.77	0.78	0.78	**0.86**	0.83	0.78	0.84	0.83	0.78
3	0.68	0.65	0.57	0.78	0.65	0.56	**0.83**	0.71	0.56
4	0.54	0.46	0.37	0.76	0.69	0.36	**0.77**	0.70	0.36
5	0.68	0.57	0.52	0.75	0.63	0.53	**0.85**	0.65	0.51
6	0.78	0.75	0.62	0.72	0.68	0.51	**0.83**	0.75	0.51
7	0.69	0.63	0.60	0.82	0.74	0.58	**0.88**	0.85	0.58
8	0.59	0.56	0.48	0.73	0.64	0.49	**0.78**	0.67	0.48

*The numbers are the AUC values for the selected models using the corresponding scoring functions. The numbers in bold font indicate the highest ranking power for top 25 models*.

### The effects of orientation mismatching

As mentioned in the previous section, the scoring functions can be reliably obtained from about 1,000 single particle scattering patterns, which are feasible to collect with the current XFEL experimental technologies. However, the results in the previous section are obtained based on a strong assumption that the orientations of the models are “exactly” matched to the orientation of native structure. It is known that orientation determination is challenging using computational methods, which utilize cross correlations between patterns by matching “experimental data” to the “model data” at discretized orientations.

**Figure 4:**
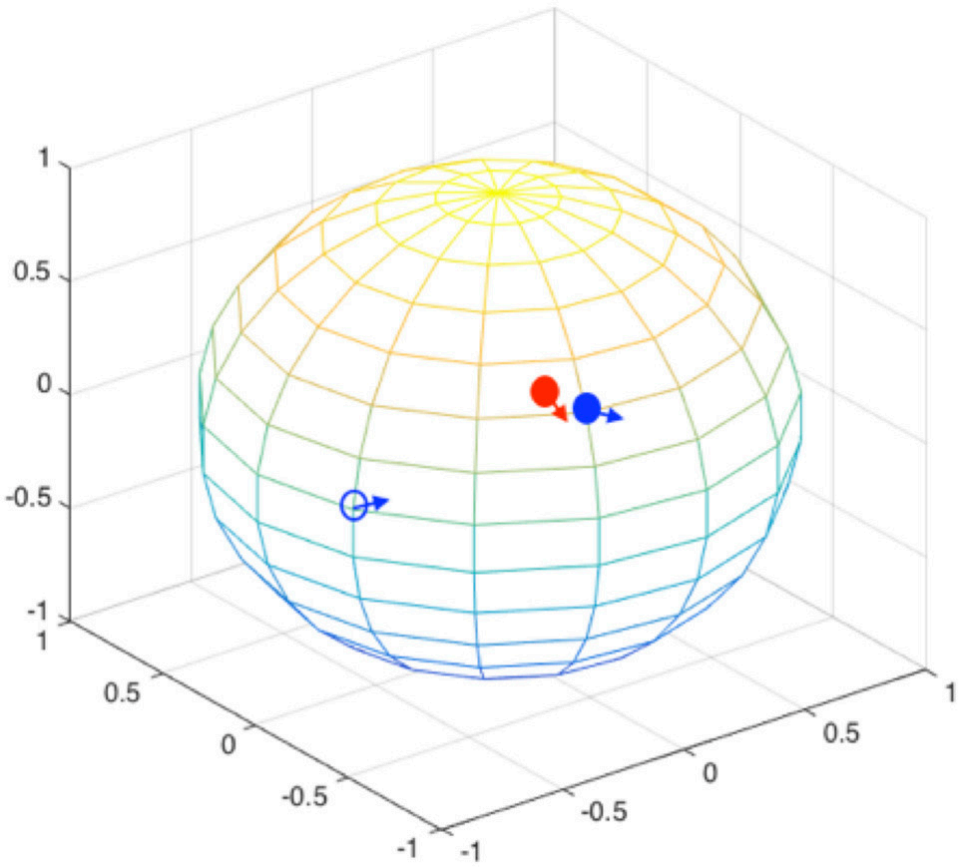
**Orientation mismatching scenarios**. Two rotation angles can be mapped to the points on a sphere, the third angle is the in-plane rotation indicated using the arrow at each point. The red solid circle and the associate arrow indicate the orientation of one experimental pattern, the blue circles and arrows indicate possible orientations. The orientation deviation of solid blue circle from the correct values (red solid circle) is due to the discretization of SO(3) rotation space; and the orientation mismatching to the open blue circle is attributed to large conformational difference. For the models that are similar to the correct complex structure, the orientations are likely to be identified to the vicinity of correct orientations (see Figure 5).

During the orientation matching, the actual orientation can be deviated from the computational matched orientation. The mismatching can happen at two levels, as schematically illustrated in Figure 4: (1) the discretized orientations for the “model” patterns are not fine enough to match the “exact” orientation but rounding up to the nearest orientations of the “exact” orientation, and this finite discretization is unavoidable due to the limitation of computing power; (2) the orientations for the best “experimental-model” pattern pairs judged by the chi-score or correlation functions are not matched, meaning that the matching is messed up by conformational differences. In this section, we implicitly considered both factors by not providing orientation information during pattern matching process. The SPI-score is calculated using the modified formula (Equation 4).

**Figure 5:**
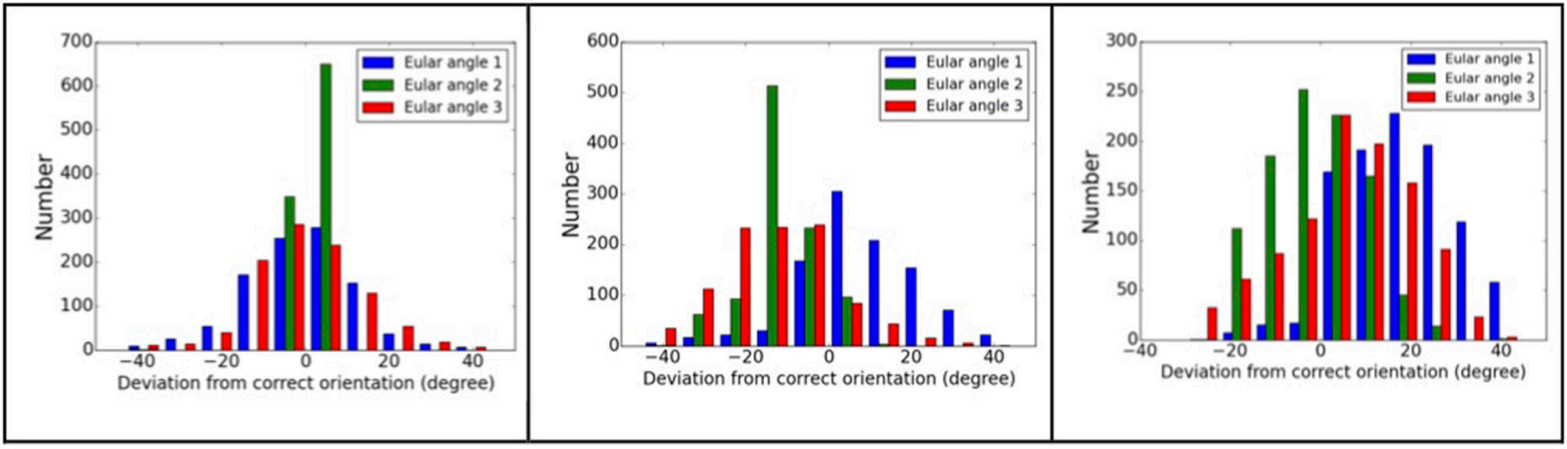
**Orientation matching results**. The dependency of matching accuracy on the conformational differences, the deviations from correct orientations for three models: left to right, the RMSD values are 2.2, 10.5, and 15.1 Å. Larger RMSD values correspond to larger deviation from correct orientations.

Using complex#1 (3AAD) as an example again, the orientation mismatching effects are studied. The matching results are summarized in Figure 5, which shows the deviation of the Euler angles from the correct orientation. For models with smaller RMSD values, most of the recovered orientations are indeed close to the orientations of “experimental” data, suggesting that the major orientation mismatching is due to the discretization of SO(3) rotation space. For the models with larger RMSD values, the success rate of determining the pattern orientations are lower, which can be explained as the consequences of conformational changes that overwhelm orientation variation effects. The statistics of the orientation deviation are summarized in Table S2. It is interesting to observe that the second rotation angle, β, is more accurately recovered using the reference matching approach than the other two angles. Using simulation data, we mapped the landscape of SPI-score due to the orientation differences. The results reveal that the SPI-score landscape around the β rotation is smoother relatively, suggesting that mismatching due to finite discretization of β angle can be tolerated. In other words, the chance of recovering the orientation within the vicinity of correct β angle is higher.

Using the subset of SO(3) rotation space, we studied the case of discretized representations of the orientations using step size of 3 degrees. The results show that the orientation matching is reasonable, and the ranking power is similar to the ideal cases discussed in the previous section. The AUC for top 25 models is 0.72 vs. 0.74 for the ideal case for complex#1 (see Table 3). Nevertheless, as the discretization step size increases, the SPI-score becomes less accurate. As a result, the ranking power of the SPI-score is reduced. When the orientation sampling is fine enough (step size of 3 degrees is sufficient in this simulation), the SPI-score outperforms the SAXS-score, which does not depend on orientation matching. The optimal discretization of SO(3) rotation space has to be chosen under the considerations of (1) the computational cost and (2) the accuracy of orientation matching. For the latter concern, the discretization step size should match the resolution of the scattering signals. For low resolution data, larger discretization step sizes can be tolerated. This may provide an opportunity of implementing multilevel model selection method to speed up the overall computing: using low resolution data to rule out a set of very unlikely models, and using higher resolutions to narrow down the best matched models.

**Table 3:** **Comparison of three methods for orientation matching**.

	**Scattering pattern**			**Radial profile**			**Correlation pattern**		
Number of selected models	1,000	100	25	1,000	100	25	1,000	100	25
AUC (RMSD)^*^	0.54	0.66	0.72	0.54	0.63	0.73	0.54	0.62	0.72
AUC (s-score)^#^	0.37	0.69	0.80	0.37	0.63	0.74	0.37	0.59	0.73
Computing time (seconds)^$^	211.47			1.07			14.30		

* *The AUC (area under curve) using RMSD as the measure for model difference*.

# *The AUC (area under curve) using s-score as the measure for model difference*.

$ *Computing time needed for orientation matching for one pattern: for raw patterns, comparing to 4,096 (16) patterns; for radial profile, comparing to 256 (16) lines; for correlation function, comparing to 256 (16) auto-correlation patterns*.

*The results are for complex#1, and the reference patterns from models are in subspace of SO(3), with discretized euler angles cover a range of [−22.5°, 22.5°] using step size of 3°*.

In order to quantify the effects of background noise to the ranking results, the signal-noise-ratio (SNR, defined as the ratio between variances of signals and noise) was varied from 100 to 0.1 logarithm spaced. The results presented in the previous sections were essentially the same with small variations in the ranking, although the absolute values of scores are larger for low SNR (i.e., larger noises for same level of signals).

### Speed up the matching of orientations

The pairwise pattern comparison requires the exhaustive sampling SO(3) rotation space using three euler angles. The pairwise 2D pattern comparison is expensive computationally, limiting the applications of this approach to large dataset. It has been found that some preprocessing of the raw scattering data can reduce computational cost for downstream analysis. First, the in-plane rotation angle can be decoupled from the other two rotations, by using an angular auto-correlation function (Huang and Liu, 2016). In this case, the computational complexity can be reduced significantly by converting the raw scattering patterns to auto-correlation functions, which are used for comparison instead of the scattering patterns. We compared the performance of the new SPI-score based on the auto-correlation functions to original SPI-score in Table 3. The results show that the ranking power is maintained to be similar, and the computational time is reduced by a factor of 14.8. Furthermore, each pattern can be reduced to a radial profile (1D) by integrating over the azimuth angle, yielding a curve that is similar to SAXS curve. Because the scores computed using the radial profile representation are essentially an average of chi-scores between matched patterns (i.e., additional information are obtained by *minimizing* the differences between experimental data and reference model), it is different from SAXS curve that is the average of radial profiles (by assuming random orientation distributions). The results show that this radial profile, although with compressed information, can be used for pairwise pattern comparisons. The score computed from radial profiles after orientation matching has a ranking power comparable to the SPI-score, as shown in Table 3. This radial profile representation further reduces the computing time by another 13.4 folds (~200 times faster than using raw pattern comparison). It is worthwhile to point out that both reduced representations do not need to sample the in-plane rotation, therefore, significantly reducing computing time of generating model patterns as well.

## Discussions

### X-rays only see electron distributions, not sequential information

**Figure 6:**
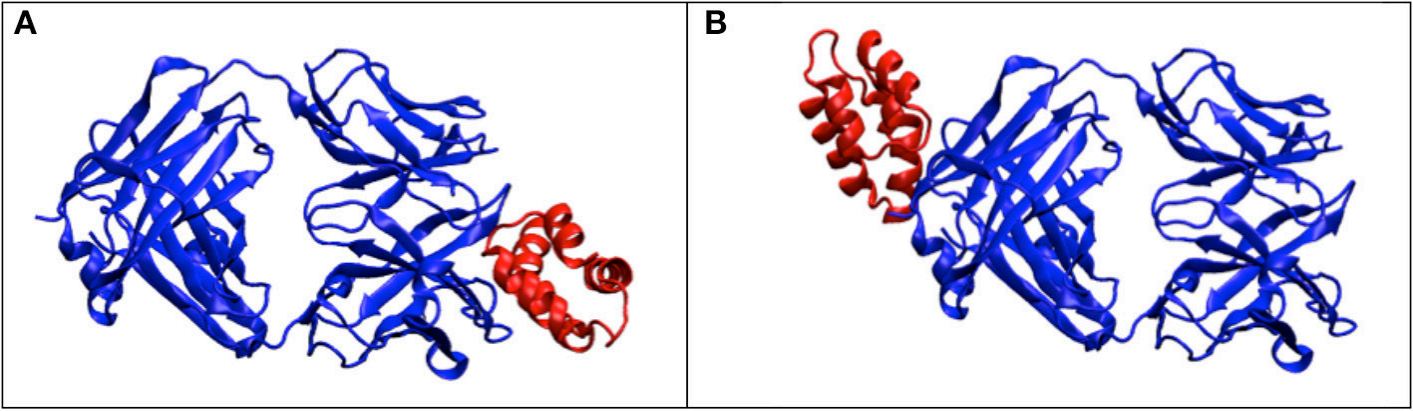
**The effects of symmetry**. The dimer complex has a pseudo-symmetry (blue color), which may reduce the model ranking power of scattering data based scoring functions. **(A)** the correct structure for the complex; **(B)** the model that has similar electron density to **(A)** after rotation, but differs significantly from **(A)** in terms of RMSD.

X-ray scattering/diffraction is due to the interaction with electrons, so the subject under probing is the electron density map. In crystallography, the atomic models are built to the electron maps by incorporating information of amino acid sequences. Without considering the sequences, the information from X-ray scattering is not sufficient to describe full features of atomic models, especially when the resolution of X-ray scattering signal is worse than atomic resolution. We observed several cases that the low SPI-scores correspond to the predicted models with large RMSD values (see Figures 3A,D). A closer examination of the corresponding models reveals that the predicted docking site is correct, but the docking pose (i.e., the orientation of the docking subunit) is opposite to the correct model. The symmetry of protein molecules can also introduce confusions in the analysis of X-ray scattering data. For example, in Figure 6, the fixed subunit molecule has a 2-fold pseudo symmetry, making it hard to distinguish the native binding modes from its symmetric counterpart. This explains some observations where the SPI-score (or SAXS-score) positively correlates to the RMSD values for models that are similar to the native structure, but the trend becomes reversed for very large RMSD values (lower SPI-scores correspond to models with larger RMSD).

**Figure 7:**
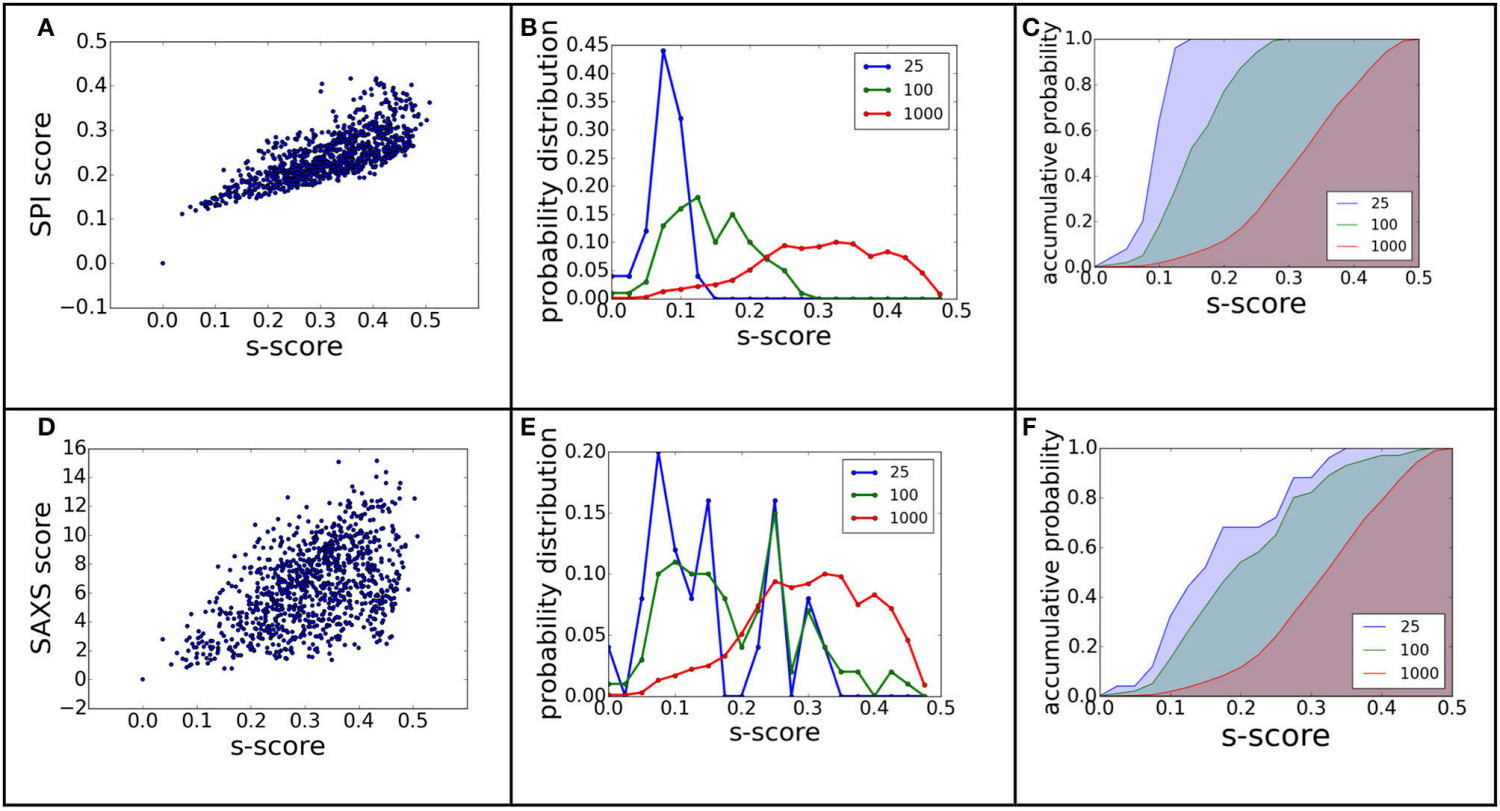
**The ranking power revisited using electron density map differences**. The figure **(A–F)** caption is the same as Figure 3, except that the model difference is measured using *s-score*, instead of RMSD.

An alternative measurement for structural differences is to treat each model as a point cloud, which ignores the sequence and connections between these points. Then, the spatial correlations between two models can be computed by maximizing their overlaps. The correlation coefficients can be calculated as the following:

(8) $$cc\;=\;\frac{\langle \rho_{1}(r)\rho_{2}(r)\rangle -\langle \rho_{1}(r)\rangle \langle \rho_{2}(r)\rangle }{\sigma_{1}\sigma_{2}}$$

where ρ_1/2_(**r**)is the electron density of model 1 or 2 at position **r**, $\sigma_{1/2}^{2}$ is the variance of model 1 or 2. We applied the model alignment method described in SASTBX programs (Liu et al., 2012). Briefly, the models are shifted such that the centers of mass coincide with the real space origin, then the relative orientations of the models are optimized by finding the largest overlaps between models. The computing is sped up by sampling three Euler angles with fast fourier transform (FFT) algorithm. In order to be consistent with RMSD that is a distance measure, we define a model difference parameter, *s-score s* = 1.0 – *cc*, to gauge the ranking power of SPI-score or SAXS-score. Using complex complex#1 (3AAD) as an example, the ranking power for scattering based scoring functions is summarized in Figure 7. The comparison to Figure 3 suggests that the X-ray scattering data is more useful in describing electron density maps. In order to compare structures that have sequential and connection information, it is necessary to incorporate knowledge of physics and chemistry. When considering the docking problem, the biochemical properties at the interface are crucial, so the model evaluation should include physicochemical terms.

### Joint scoring function is needed to outperform individual functions

We examined the relation between SPI-scores and the SAXS-scores by computing the correlation coefficients (See Table S3 in Supplementary Material). The results suggest positive correlation between the two scoring functions, with varying correlation strength (0.12 to 0.81). This variation suggests that the two scoring functions contain different structural information. As shown in the Result section, the SPI-score is better in ranking the models, so it is natural to include the SPI-score in the joint scoring function.

The built-in scoring function of Z-dock is not sufficient in ranking the models, but it has its merit by design, which incorporates physicochemical terms and geometry complementary properties. The model ranking by each scoring approach is unlikely to outperform the combined scores. The optimized IRAD (integration of residue- and atom-based potentials for docking) function was reported to improve the model ranking by combining several scoring functions (Vreven et al., 2011). We re-ranked the models using z-rank program where IRAD functions are implemented (Pierce and Weng, 2007). However, the model ranking power is increased modestly in this case, mainly because the Z-dock program has a built-in scoring function that give comparable ranking power as IRAD scores.

In order to explore the potential of joint scoring functions, we experimented one method of combination using SPI-score and Z-dock score using a voting system: first, the Z-scores are calculated for each model with either SPI-score or Z-dock score, then the Z-scores are combined to give an overall ranking. The experiment for complex model (#1) dataset does not yield significant improvement. This suggests that it is not trivial to combine the scores from different evaluation methods, because hybrid does not mean simple linear combination. Designing better ways to combine different scoring functions are subjects of future studies.

### Hybrid approach can be applied to incomplete dataset

Although the idea in this work is about applying experimental data in SPI or SAXS in the ranking of docking models, the impact of modeling to the data interpretation is equally significant. As mentioned, the XFEL facilities are scarce resources, although more XFEL facilities will be commissioned in the near future, there are still some technological challenges to carry out high throughput single particle scattering experiments. It is not practical to collect complete datasets for model reconstructions that are solely based on experimental data yet. If computational modeling, such as molecular docking or protein structure prediction, is integrated in the data interpretation, it is possible to determine structures from a much smaller dataset (~1,000 patterns in the simulation cases). In other words, the hybrid approach turns a reverse modeling (from intensity to electron density map) problem to a ranking problem of the predicted models. Given the advances in high performance computing, sampling algorithms will be capable of generating diverse models, in which the correct structure is very likely to be included. Then the model ranking and selection criteria is the key to model determination.

In a related research field, the cryogenic electron microscopy (CryoEM), the projection images of molecules are detected. Several algorithms have been developed to reconstruct detailed 3D structures based on projection images. In general, such dataset must be composed of a large number of images (at the order of 10 thousands to 100 thousands), in order to obtain high resolution structures. For relative low resolution model reconstruction, it is feasible to obtain an *ab initio* density map with <1,000 patterns using the maximum likelihood method (Ekeberg et al., 2015). A global assignment of orientations is also reported for simulation data using common line algorithm for fewer than 1,000 patterns (Singer and Shkolnisky, 2011). The hybrid approach reported here can potentially be used to select the models at higher resolutions with similar amount of data, given the availability of high resolution structures of docking subunits.

## Conclusion

The development of XFEL and its application in single particle imaging requires fast and reliable methods to interpret experimental data, especially when the dataset is not sufficient to convert scattering signals to a unique structural model. In this work, we demonstrated that single particle experimental data is valuable in ranking the predicted models, and this hybrid approach can be one solution for structure determination with limited XFEL data.

## Author contributions

HL designed the research, HW carried the simulations, both authors analyzed the data and contributed to the manuscript writing.

### Conflict of interest statement

The authors declare that the research was conducted in the absence of any commercial or financial relationships that could be construed as a potential conflict of interest.
