# Supplementary material for: Determining Complex Structures using Docking Method with Single Particle Scattering Data
Source: Front Mol Biosci. 2017 Apr 25;4:23. doi: 10.3389/fmolb.2017.00023 (PMC5403940; doi:10.3389/fmolb.2017.00023)
Supplement: Supplementary file 1 [file DataSheet1.docx]

Supplementary Materials for

Determining complex structures using docking method with single particle scattering data

Hongxiao Wang^1^, Haiguang Liu^1*^

^1^Complex Systems Division, Beijing Computational Science Research Center, 10 West Dongbeiwang Rd, Shangdi, Beijing 100193, China

*** Correspondence:**Haiguang Liu
hgliu@csrc.ac.cn

The native structure of complexes used in the article can be accessed from the website: <http://liulab.csrc.ac.cn>. The decoy sets can be generated using Zdock program or requested from the authors.

Table S1. Simulation Parameters

| Detector Size | 129 x 129 pixels |
| --- | --- |
| Pixel Size | 397 x 397 micron |
| Distance between sample and Detector | 1 m |
| Wavelength (λ) | 1Å |
| Resolution at edge of detector | 4Å |

Table S2. Statistics of angle mismatching.

|  | Native | Complex 1 | Complex 2 | Complex 3 |
| --- | --- | --- | --- | --- |
| RMSD* (Å) | 0 | 2.2 | 10.7 | 15.3 |
| α – α0 (**°)** | 0.3 (5.6) | -0.5 (12.7) | 8.1 (14.1) | 17.7 (12.1) |
| β – β0 (**°)** | 0.02 (0.87) | 1.0 (1.0) | -12.3 (8.8) | -1.0 (10.3) |
| γ – γ0 (**°)** | 0.3 (5.7) | -0.3 (12.5) | 5.6 (13.6) | -12.9 (13.8) |

Symbols:

*RMSD is the Root-Mean-Square-Deviation with a unit of Å, compared to the native complex structure.

(α,β,γ) are the three euler angles by matching the ‘experimental’ patterns to the reference patterns generated from either native structure or decoy models (complex 1, 2, 3). (α0,β0,γ0) are the euler angles used for the simulation of ‘experimental’ patterns. The numbers are the mean value of the angle mismatching with the standard deviation in the parenthesis.

Table S3. Correlation between SAXS-score and SPI-score.

| Complex | Correlation coefficient (SPI-score, SAXS-score) |
| --- | --- |
| #1 | 0.36 |
| #2 | 0.78 |
| #3 | 0.80 |
| #4 | 0.81 |
| #5 | 0.12 |
| #6 | 0.44 |
| #7 | 0.56 |
| #8 | 0.81 |

Eight complex systems were used to systematically study the ranking power of scattering based scoring functions.

Figure S1. The scatter plot, the RMSD distribution function, and accumulative distribution function are shown. For each complex, the upper panels are SPI_scores, and lower panels are the SAXS_scores, and the structure differences are measured using RMSD.

| 1 | 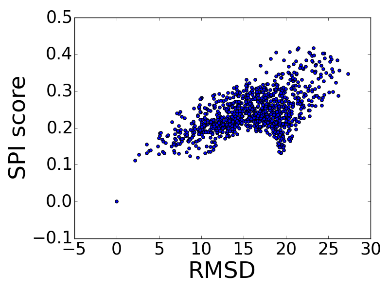 | 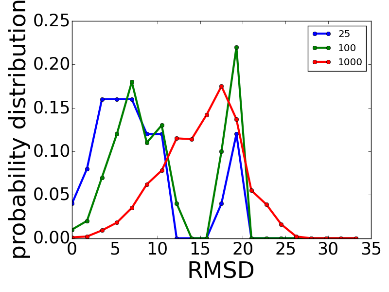 | 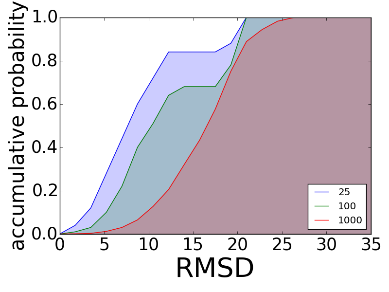 |
| --- | --- | --- | --- |
|  | 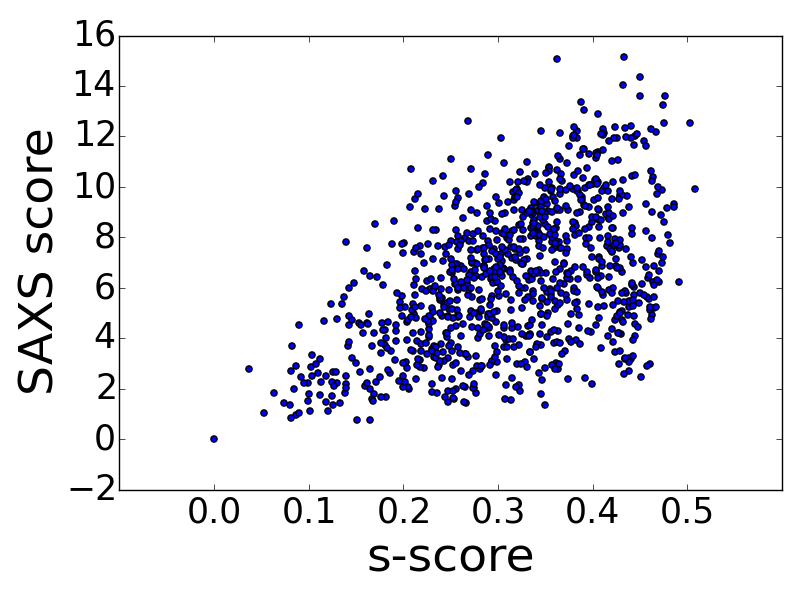 | 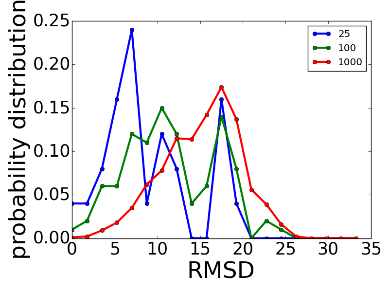 | 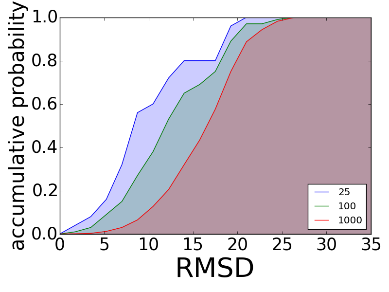 |
| 2 | 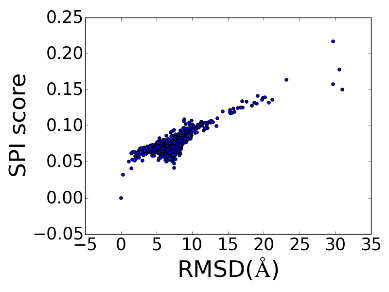 | 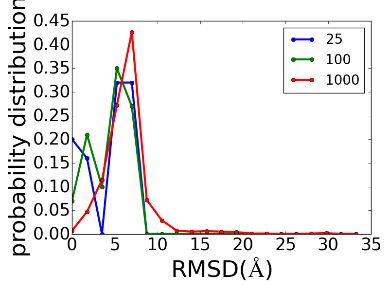 | 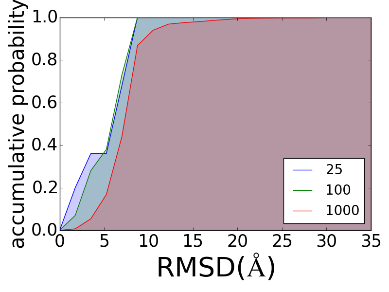 |
|  | 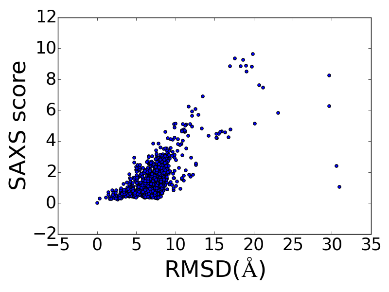 | 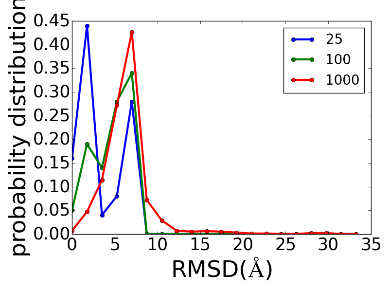 | 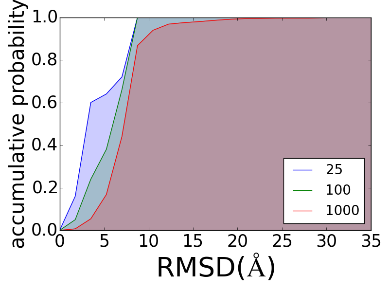 |
| 3 | 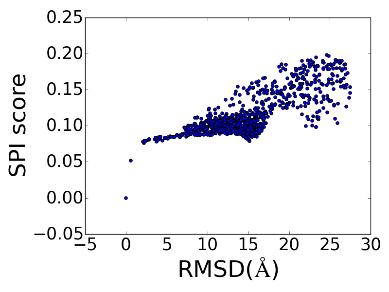 | 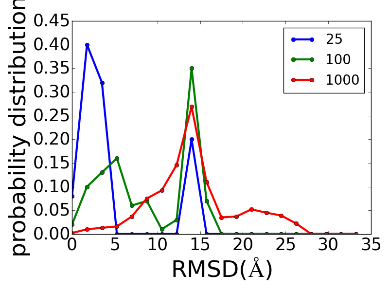 | 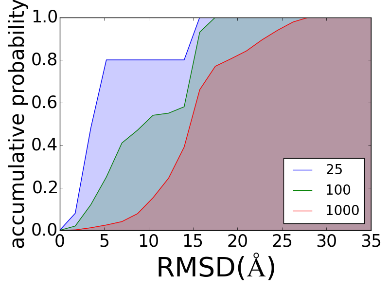 |
|  | 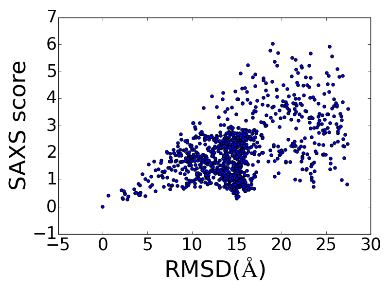 | 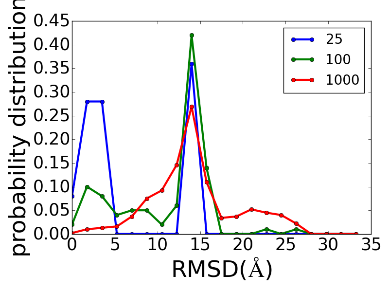 | 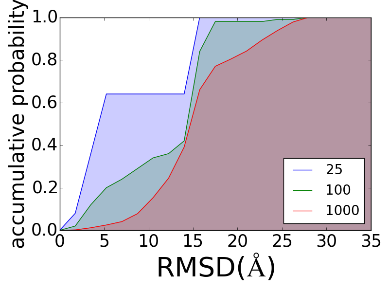 |
| 4 | 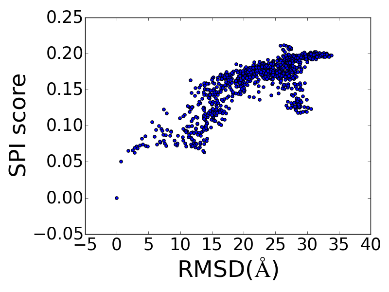 | 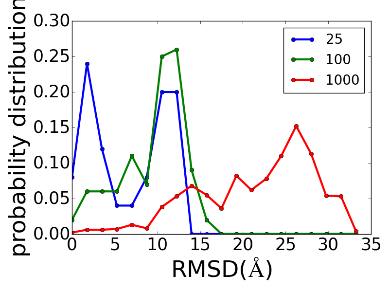 | 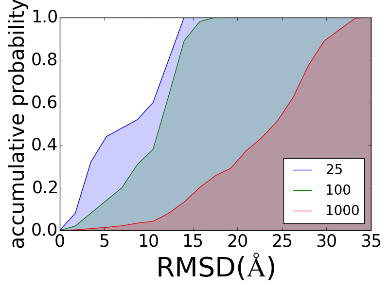 |
|  | 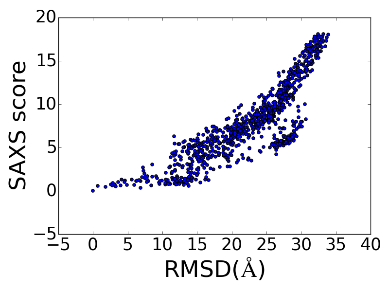 | 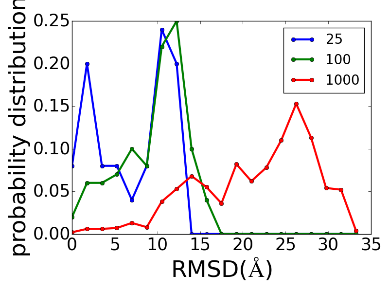 | 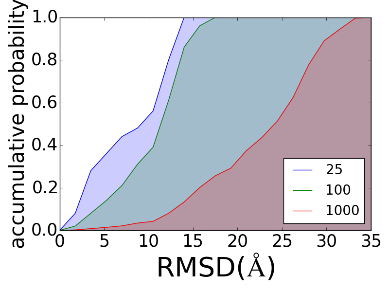 |
| 5 | 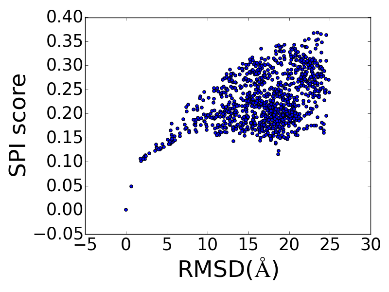 | 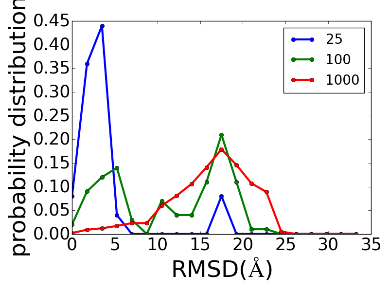 | 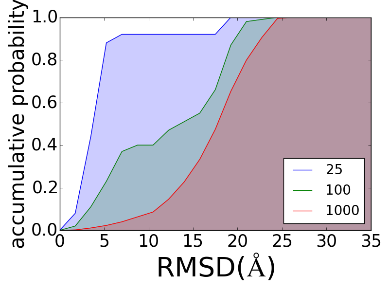 |
|  | 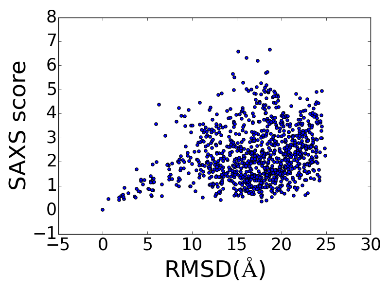 | 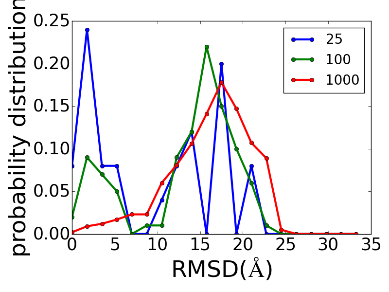 | 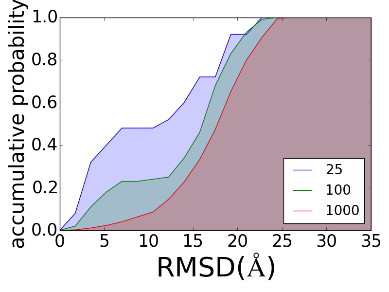 |
| 6 | 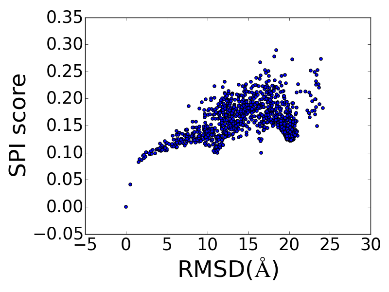 | 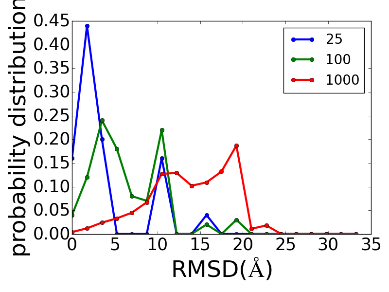 | 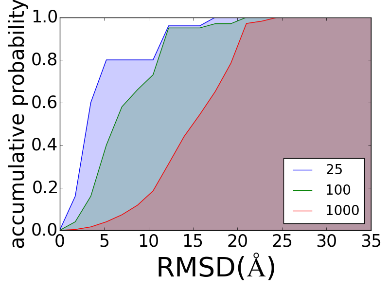 |
|  | 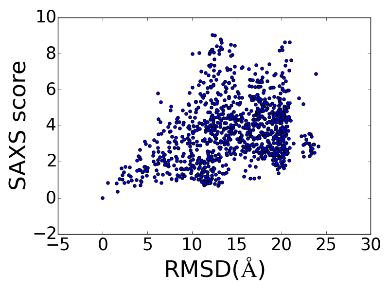 | 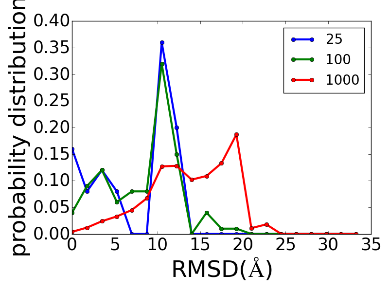 | 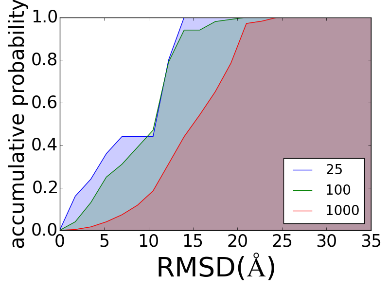 |
| 7 | 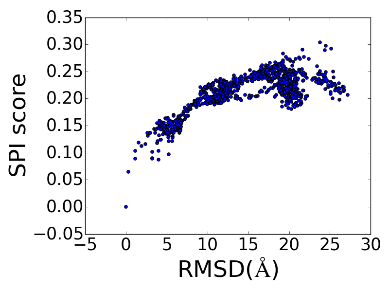 | 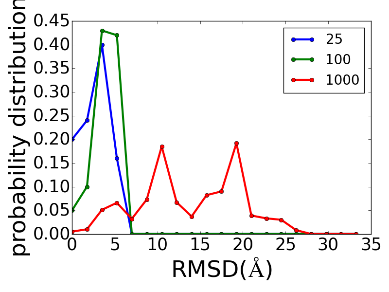 | 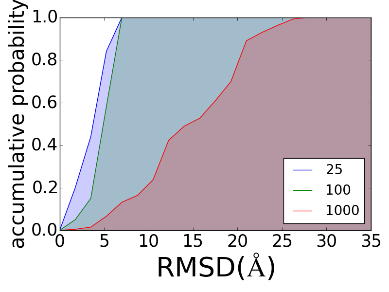 |
|  | 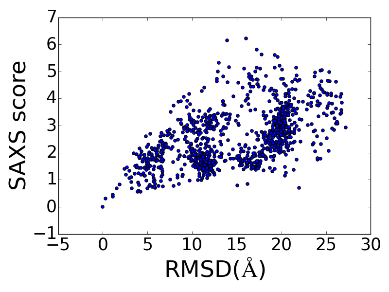 | 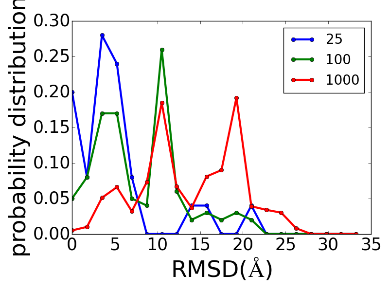 | 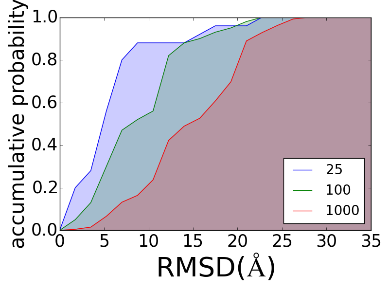 |
| 8 | 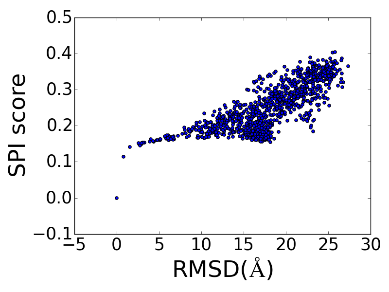 | 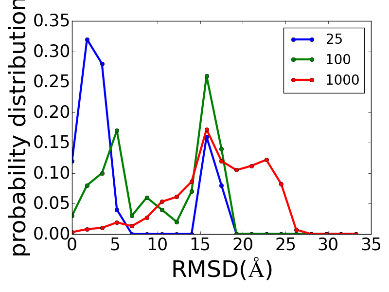 | 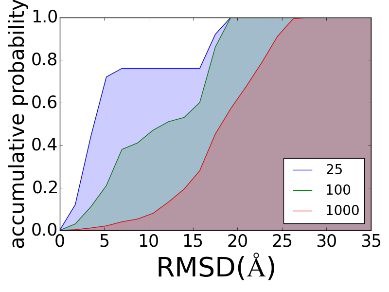 |
|  | 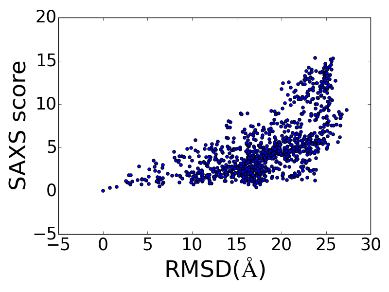 | 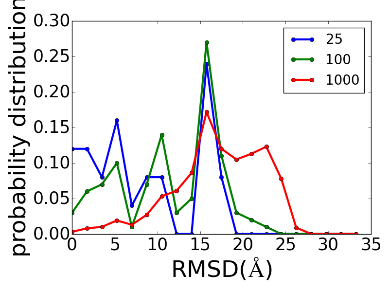 | 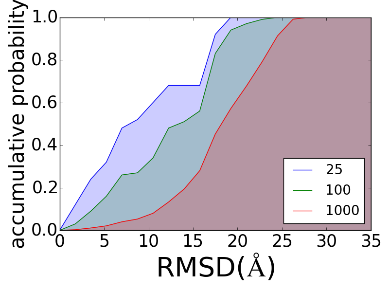 |

Figure S2. Same as in S1, but the model difference is measured using the s-score defined in the main text. The scatter plot, the s-score distribution function, and accumulative distribution function are shown. For each complex, the upper panels are SPI_scores, and lower panels are the SAXS_scores.

| 1 | 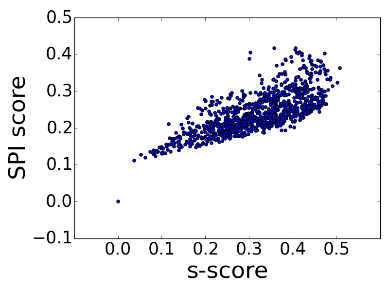 | 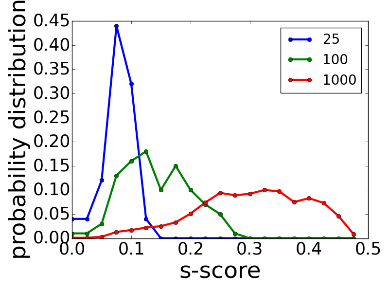 | 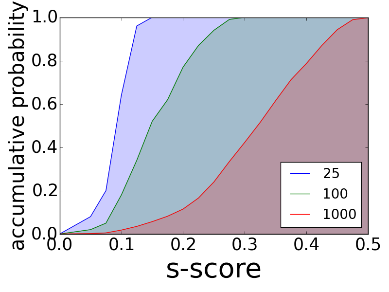 |
| --- | --- | --- | --- |
|  | 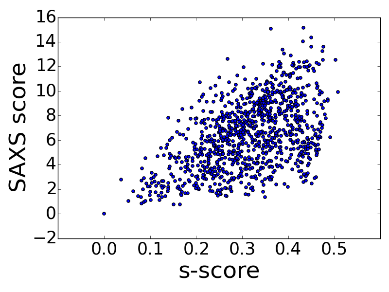 | 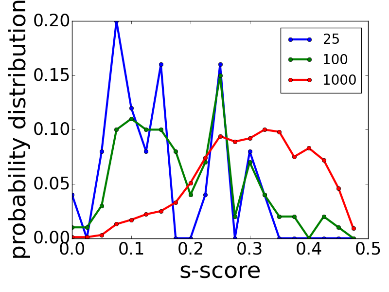 | 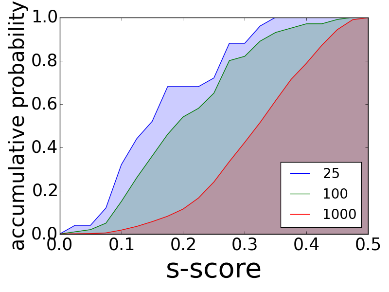 |
| 2 | 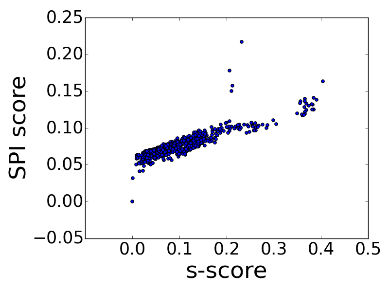 | 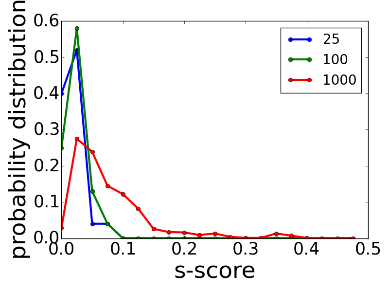 | 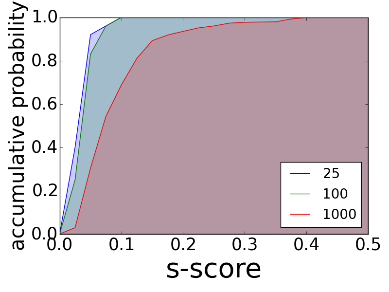 |
|  | 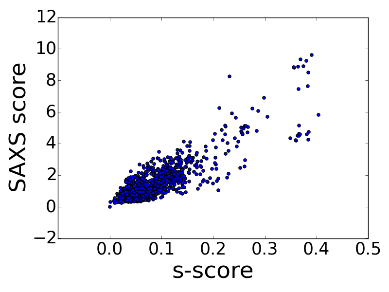 | 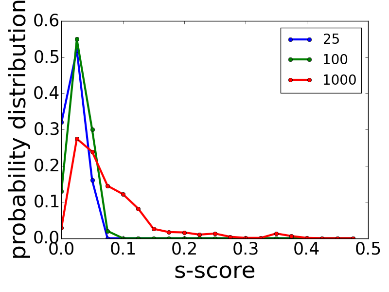 | 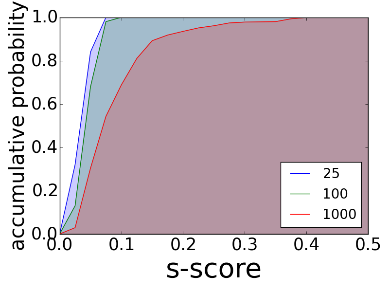 |
| 3 | 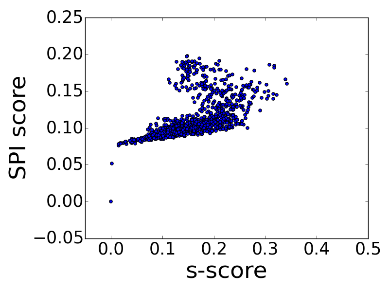 | 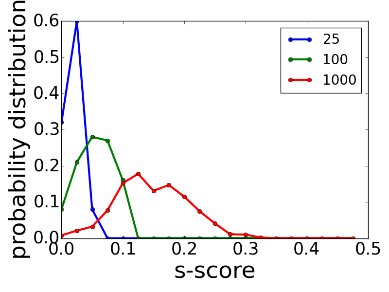 | 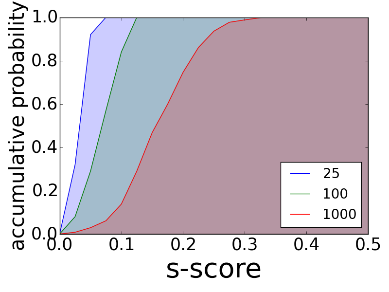 |
|  | 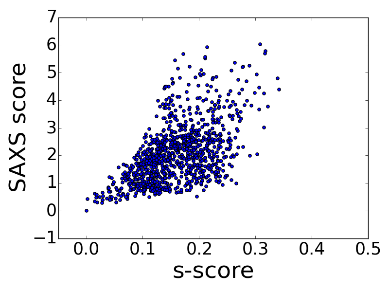 | 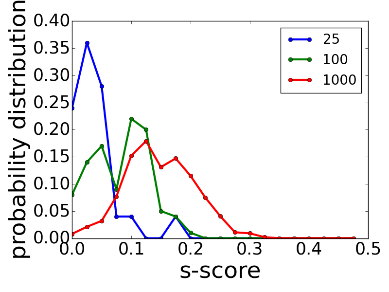 | 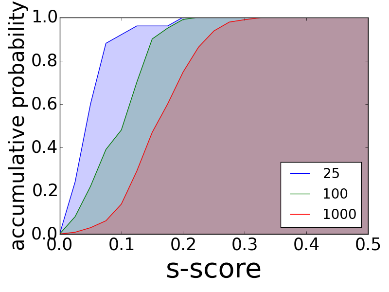 |
| 4 | 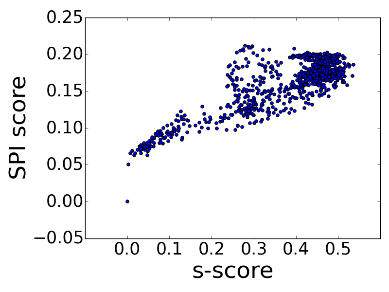 | 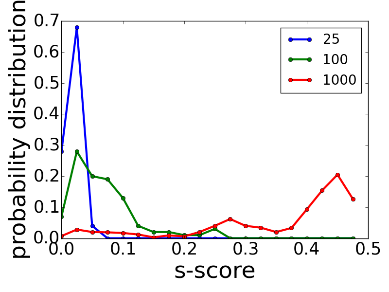 | 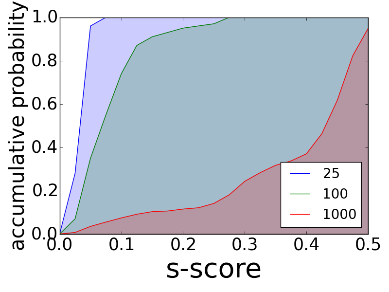 |
|  | 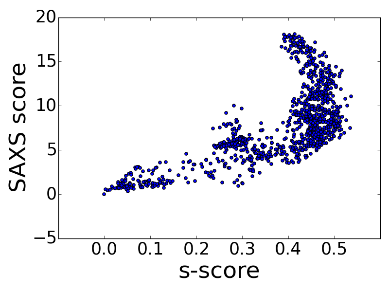 | 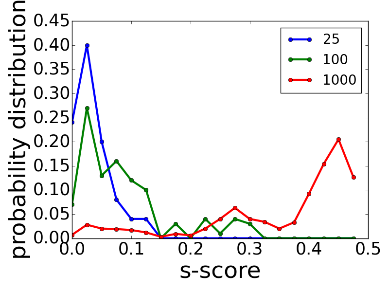 | 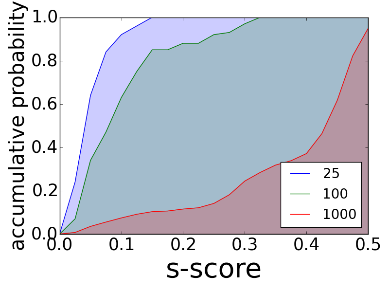 |
| 5 | 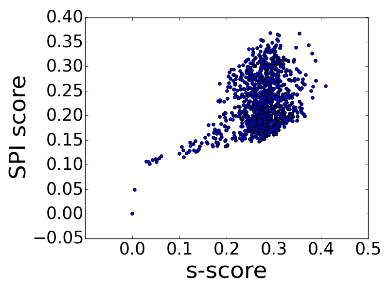 | 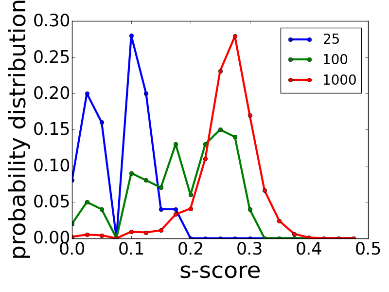 | 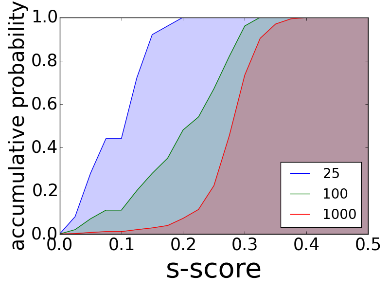 |
|  | 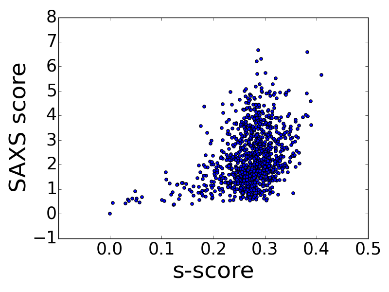 | 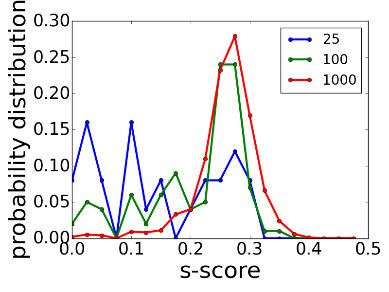 | 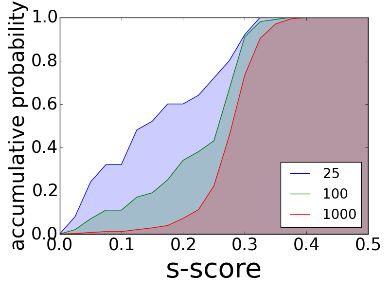 |
| 6 | 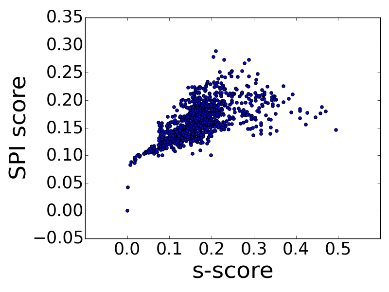 | 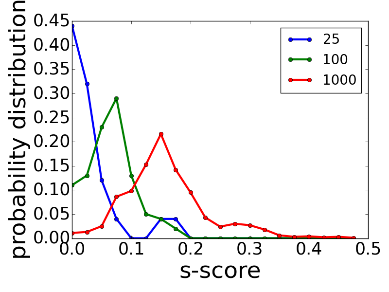 | 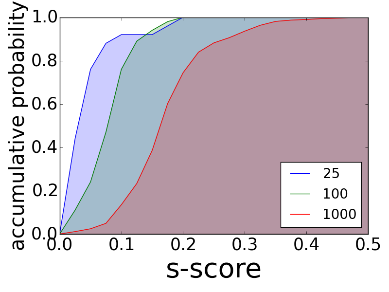 |
|  | 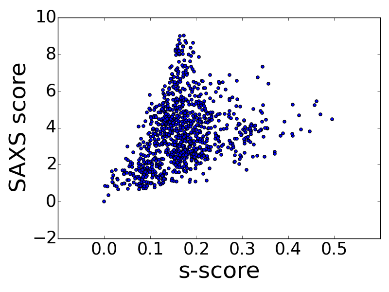 | 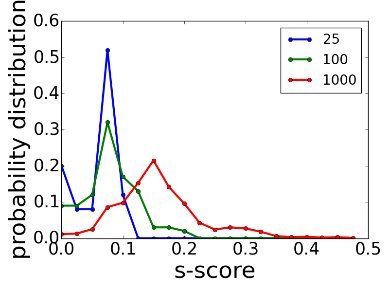 | 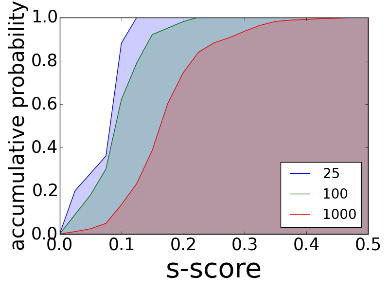 |
| 7 | 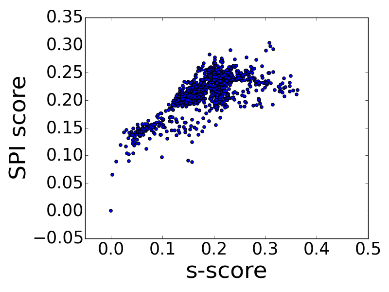 | 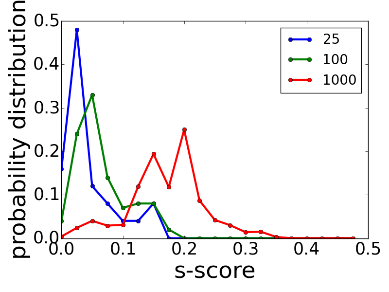 | 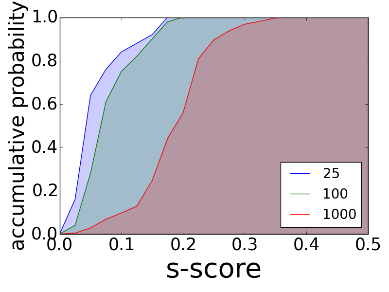 |
|  | 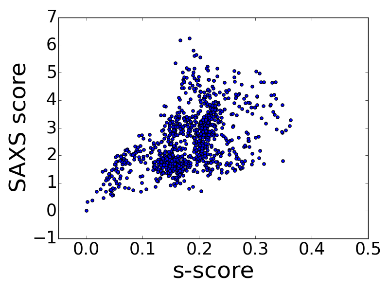 | 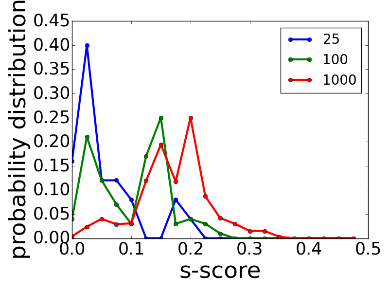 | 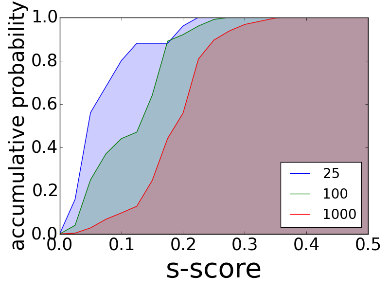 |
| 8 | 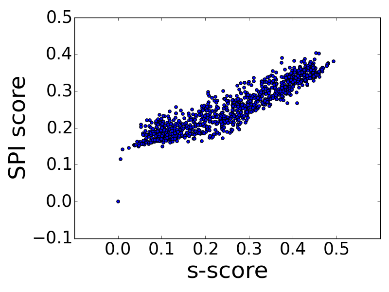 | 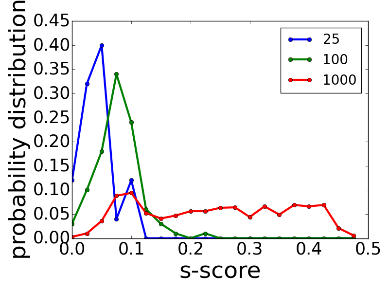 | 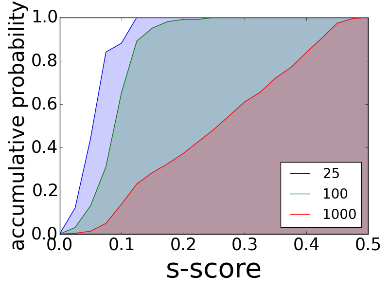 |
|  | 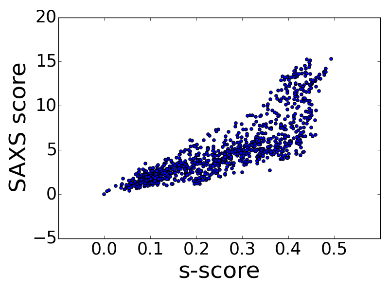 | 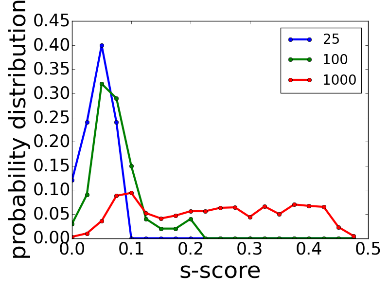 | 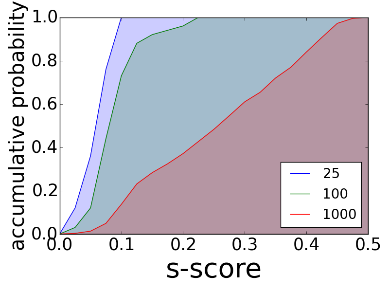 |
